# Association between smoking behavior and serum uric acid among the adults: Findings from a national cross-sectional study

**DOI:** 10.1371/journal.pone.0285080

**Published:** 2023-05-02

**Authors:** Yun Seo Jang, Nataliya Nerobkova, Il Yun, Hyunkyu Kim, Eun-Cheol Park

**Affiliations:** 1 Department of Public Health, Graduate School, Yonsei University, Seoul, Republic of Korea; 2 Institute of Health Services Research, Yonsei University, Seoul, Republic of Korea; 3 Department of Preventive Medicine, Yonsei University College of Medicine, Seoul, Republic of Korea; Shahid Beheshti University of Medical Sciences, ISLAMIC REPUBLIC OF IRAN

## Abstract

**Background:**

Gout incidence is increasing worldwide; appropriate management of serum uric acid levels and a healthy lifestyle may help its prevention. The popularity of electronic cigarettes and the resultant emergence of dual smokers is increasing. Despite many studies on the effects of various health behaviors on serum uric acid levels, the association between smoking and serum uric acid levels remains controversial. This study aimed to investigate the association between smoking and serum uric acid levels.

**Methods:**

In this study, total sample of 27,013 participants (11,924 men and 15,089 women) were analyzed. This study used data from the Korea National Health and Nutrition Examination Survey (2016–2020) and grouped adults into dual smokers, single smokers, ex-smokers, and non-smokers. Multiple logistic regression analyses were performed to investigate the association between smoking behavior and serum uric acid levels.

**Results:**

Compared to male non-smokers, male dual smokers had significantly higher serum uric acid level (odds ratio [OR], 1.43; 95% confidence interval [CI], 1.08–1.88). In female, serum uric acid level was higher among single smokers than non-smokers (OR, 1.68; 95% CI, 1.25–2.25). Higher serum uric acid levels were more likely to be present in male dual smokers with a > 20 pack-year smoking habit (OR, 1.84; 95% CI, 1.06–3.18).

**Conclusion:**

Dual smoking may contribute to high serum uric acid levels in adults. Thus, serum uric acid levels should be properly managed through smoking cessation.

## Introduction

Gout is a type of auto-inflammatory arthritis with increasing prevalence and incidence worldwide [1, 2]. Increased incidence and death rate have been reported especially in the United States, Italy, South Korea, Australia, New Zealand, and Taiwan [3, 4]. Serum uric acid (SUA) plays a pivotal role in gout and is an unusual complication of anorexia nervosa [5]. Moreover, SUA is a potential risk factor for the deterioration of kidney function; high SUA levels increase the risk of acute and chronic kidney disease (CKD) [6]. High SUA levels progress to hyperuricemia, which may play a role in the pathogenesis of CKD and damage the vascular lining over time [7]. Additionally, SUA is associated with other health risk factors in daily life, such as hypertension, insulin resistance, and the cardiovascular diseases [8–10]. Given that elevated SUA causes many diseases in the contemporary world, its management is significant for personal health [11].

Smoking is a leading risk factor for premature death worldwide and is one of the primary causes of chronic diseases such as cancer, cardiovascular diseases, and respiratory diseases [12]. Owing to the adverse health outcomes of smoking, people tend to quit conventional smoking and are increasingly turning to electronic cigarettes (e-cigarettes) as an alternative [13, 14]. E-cigarettes heat a liquid that often contains nicotine to produce aerosol, which is subsequently inhaled. Evidence reveals that dual smoking behavior, which involves smoking both e-cigarettes and conventional cigarettes, is as harmful to health as smoking conventional cigarettes alone [15–19]. Although e-cigarette nicotine delivery systems are considered less dangerous than conventional cigarettes, they are associated with a range of complications, including thermal damage, lung damage, cardiovascular outcomes, and psychosocial effects [18]. As of 2020, Korea’s smoking rate is 20.6% (male: 34.0%, female: 6.6%), of which 8.4% for male and 1.9% for female use e-cigarettes. With an increase in the number of dual smokers and decrease in successful smoking cessation observed, dual smoking appears to have the potential to induce tobacco dependence [20–23].

Previous SUA level-related studies have found associations with gout, kidney function, alcohol, tea, coffee, milk, and yogurt [7, 8]. Notably, in the United States, patients with gout are advised to limit the consumption of distilled beverages such as beer and wine and are recommended to consume low-fat or non-fat dairy products [10]. Despite studies conducted by various research groups on the association between smoking and SUA, many conflicting opinions still exist [24]. Additionally, unlike the evidence related to smoking, there is insufficient evidence to clarify the association between e-cigarette or dual smoking and SUA.

Therefore, this study aimed to investigate the association between various smoking behaviors, including dual smoking (both e-cigarettes and conventional cigarettes), single smoking (only conventional cigarettes), and past smoking with respect to SUA, in a representative Korean adult population.

## Materials and methods

### Data and study population

The data used in this study were obtained from the Korea National Health and Nutrition Examination Survey (KNHANES) conducted from 2016 to 2020. The KNHANES is a cross-sectional, nationwide survey conducted annually by the Korea Disease Control and Prevention Agency (KDCA) of the Ministry of Health and Welfare, South Korea, to evaluate the health status, health behavior, and nutritional status of the South Korean population. The respondents answered the questionnaires, and all the obtained data were anonymized. As the KNHANES complies with the Declaration of Helsinki and provides publicly accessible data, ethical approval was not required.

The total number of respondents from the 2016–2020 survey was 39,738. Information from individuals aged 1–18 years was excluded as they had not been asked regarding smoking behavior (*N* = 7,610). Additionally, data from participants with missing variables were also excluded (*N* = 5,105). Finally, 27,013 participants (11,924 men and 15,089 women) were analyzed in this study.

### Measures

The dependent variable, SUA was measured by collecting venous blood from participants who had been fasting for > 8 h. SUA was measured by colorimetry with the enzyme uricase using a Hitachi Automatic Biochemical Analyzer 7600–210 (Hitachi, Tokyo, Japan) from 2016 to 2018 in KNHANES. Uricase was also measured using Labospect 008AS (Hitachi, Tokyo, Japan) from 2019 to 2020 in KNHANES. Furthermore, the common cutoff value for SUA level was 7.0 mg/dL (420 μmol/L) for men and 6.0 mg/dL (357 μmol/L) for women [25].

Based on smoking behavior as the independent variable, the study population was divided into four groups: (1) non-smokers, (2) ex-smokers who had been using conventional cigarettes or e-cigarettes in the past, (3) single smokers who used only conventional cigarettes, and (4) dual smokers who used conventional cigarettes and e-cigarettes. This classification was identical to that of the previous studies that investigated smoking behavior using the same investigative tools [16, 17, 26].

The covariates included demographic factors: age (19–29 / 30–39 / 40–49 / 50–59 / 60–69 / ≥ 70), marital status (married / single or widow / divorced or separated), and educational level (middle school or below / high school / college or over); socioeconomic factors: household income (low / mid-low / mid-high / high), region of residence (metropolitan / urban / rural), and occupation (white / pink / blue / inoccupation); health-related factors: body mass index [BMI] (underweight / normal / overweight), hypertension status (normal / pre-hypertension / hypertension), diabetes status (yes / no), and dyslipidemia status (yes / no); and health-related behavioral patterns of alcohol consumption (yes / no).

### Statistical analysis

All estimates were calculated using sample weight procedures, clusters, and strata assigned to the study participants. Descriptive analysis was performed to assess the general characteristics of the study population. Subsequently, a multiple logistic regression analysis was performed to evaluate the effect of smoking behavior on SUA levels and perform a subgroup analysis stratified by independent variables. In addition, we calculated the pack-years by the amount and duration of smoking in the past or current smokers and conducted a subgroup analysis by tying it with the smoking behavior. The main results are expressed as odds ratios (ORs) and 95% confidence intervals (CIs). SAS version 9.4 (SAS Institute Inc.; Cary, NC, USA) was used for all analyses, and a p-value <0.05 was considered statistically significant.

## Results

Table 1 highlights the general characteristics of the study population. Of the 27,013 participants, 11,924 were men (44.1%) and 15,089 were women (55.9%). Among the men, 357 (3.0%), 3,629 (30.4%), 5,057 (42.4%), and 2,881 (24.2%) were dual smokers, single smokers, ex-smokers, and non-smokers, respectively. Among the women, 72 (0.5%), 697 (4.6%), 938 (6.2%), and 13,382 (88.7%) were dual smokers, single smokers, ex-smokers, and non-smokers, respectively. The relationship between smoking behavior and SUA levels was statistically significant in men and women. Moreover, differences in demographic, socioeconomic, and health status characteristics were primarily significant (p < .0001).

**Table 1:** General characteristics of the study population.

Variables		Male							Female						
		Serum Uric Acid Level							Serum Uric Acid Level						
		Total		normal (<7)		abnormal (≥7)		*P-value*	Total		normal (<6)		abnormal (≥6)		*P-value*
		N	%	N	%	N	%		N	%	N	%	N	%	
**Total (*N* = 27,013)**		11,924	100.0	9,482	79.5	2,442	20.5		15,089	100.0	13,953	92.5	1,136	7.5	
**Smoking Behavior**								< .0001							< .0001
	Non-smoker	2,881	24.2	2,286	79.3	595	20.7		13,382	88.7	12,412	92.8	970	7.2	
	Ex-smoker	5,057	42.4	4,069	80.5	988	19.5		938	6.2	862	91.9	76	8.1	
	Single smoker	3,629	30.4	2,884	79.5	745	20.5		697	4.6	615	88.2	82	11.8	
	Dual smoker	357	3.0	243	68.1	114	31.9		72	0.5	64	88.9	8	11.1	
**Age**								< .0001							< .0001
	19–29	1,636	13.7	1,169	71.5	467	28.5		1,757	11.6	1,634	93.0	123	7.0	
	30–39	1,858	15.6	1,327	71.4	531	28.6		2,297	15.2	2,158	93.9	139	6.1	
	40–49	2,146	18.0	1,653	77.0	493	23.0		2,852	18.9	2,726	95.6	126	4.4	
	50–59	2,204	18.5	1,857	84.3	347	15.7		2,999	19.9	2,805	93.5	194	6.5	
	60–69	2,126	17.8	1,833	86.2	293	13.8		2,743	18.2	2,540	92.6	203	7.4	
	≥70	1,954	16.4	1,643	84.1	311	15.9		2,441	16.2	2,090	85.6	351	14.4	
**Marital status**								< .0001							< .0001
	Married	8,548	71.7	6,965	81.5	1,583	18.5		10,064	66.7	9,434	93.7	630	6.3	
	Single, widow	2,861	24.0	2,097	73.3	764	26.7		4,145	27.5	3,716	89.7	429	10.3	
	Divorced, Separated	515	4.3	420	81.6	95	18.4		880	5.8	803	91.3	77	8.8	
**Educational level**								< .0001							< .0001
	Middle school or below	2,698	22.6	2,261	83.8	437	16.2		4,994	33.1	4,459	89.3	535	10.7	
	High school	4,209	35.3	3,338	79.3	871	20.7		4,730	31.3	4,416	93.4	314	6.6	
	College or over	5,017	42.1	3,883	77.4	1,134	22.6		5,365	35.6	5,078	94.7	287	5.3	
**Household income**								0.0260							< .0001
	Low	1,916	16.1	1,563	81.6	353	18.4		2,922	19.4	2,583	88.4	339	11.6	
	Mid-low	2,850	23.9	2,260	79.3	590	20.7		3,707	24.6	3,409	92.0	298	8.0	
	Mid-high	3,375	28.3	2,699	80.0	676	20.0		4,108	27.2	3,865	94.1	243	5.9	
	High	3,783	31.7	2,960	78.2	823	21.8		4,352	28.8	4,096	94.1	256	5.9	
**Region**								0.1921							0.0005
	Metropolitan	5,196	43.6	4,125	79.4	1,071	20.6		6,756	44.8	6,293	93.1	463	6.9	
	Urban	4,421	37.1	3,492	79.0	929	21.0		5,599	37.1	5,177	92.5	422	7.5	
	Rural	2,307	19.3	1,865	80.8	442	19.2		2,734	18.1	2,483	90.8	251	9.2	
**Occupational categories**								< .0001							< .0001
	White	3,496	29.3	2,644	75.6	852	24.4		3,390	22.5	3,214	94.8	176	5.2	
	Pink	1,225	10.3	946	77.2	279	22.8		2,295	15.2	2,159	94.1	136	5.9	
	Blue	3,908	32.8	3,225	82.5	683	17.5		2,272	15.1	2,107	92.7	165	7.3	
	Inoccupation	3,295	27.6	2,667	80.9	628	19.1		7,132	47.3	6,473	90.8	659	9.2	
**BMI**								< .0001							< .0001
	Underweight	284	2.4	259	91.2	25	8.8		718	4.8	710	98.9	8	1.1	
	Normal	6,639	55.7	5,602	84.4	1,037	15.6		9,827	65.1	9,325	94.9	502	5.1	
	Overweight	5,001	41.9	3,621	72.4	1,380	27.6		4,544	30.1	3,918	86.2	626	13.8	
**Alcohol consumption**								< .0001							0.0007
	Yes	9,883	82.9	7,762	78.5	2,121	21.5		9,792	64.9	9,107	93.0	685	7.0	
	No	2,041	17.1	1,720	84.3	321	15.7		5,297	35.1	4,846	91.5	451	8.5	
**Status of Hypertension**								< .0001							< .0001
	Normal	4,048	33.9	3,363	83.1	685	16.9		7,585	50.3	7,237	95.4	348	4.6	
	Pre-Hypertension	3,647	30.6	2,839	77.8	808	22.2		3,110	20.6	2,895	93.1	215	6.9	
	Hypertension	4,229	35.5	3,280	77.6	949	22.4		4,394	29.1	3,821	87.0	573	13.0	
**Status of Diabetes**								< .0001							< .0001
	Yes	1,238	10.4	1,051	84.9	187	15.1		1,000	6.6	850	85.0	150	15.0	
	No	10,686	89.6	8,431	78.9	2,255	21.1		14,089	93.4	13,103	93.0	986	7.0	
**Status of Dyslipidemia**								0.3910							< .0001
	Yes	2,503	21.0	1,975	78.9	528	21.1		3,955	26.2	3,565	90.1	390	9.9	
	No	9,421	79.0	7,507	79.7	1,914	20.3		11,134	73.8	10,388	93.3	746	6.7	
**Year**								0.0190							0.0004
	2016	2,332	19.6	1,894	81.2	438	18.8		3,064	20.3	2,851	93.0	213	7.0	
	2017	2,430	20.4	1,959	80.6	471	19.4		3,016	20.0	2,830	93.8	186	6.2	
	2018	2,428	20.4	1,916	78.9	512	21.1		3,126	20.7	2,872	91.9	254	8.1	
	2019	2,448	20.5	1,901	77.7	547	22.3		3,113	20.6	2,834	91.0	279	9.0	
	2020	2,286	19.2	1,812	79.3	474	20.7		2,770	18.4	2,566	92.6	204	7.4	

Table 2 presents the association between smoking behavior and SUA levels in men and women after adjusting for all covariates. In men, dual smokers (OR, 1.43; 95% CI, 1.08–1.88) were statistically associated with SUA, whereas in women, a statistical association was observed in single smokers (OR, 1.68; 95% CI, 1.25–2.25). Compared with non-smokers, both male and female ex-smokers, single smokers, and dual smokers showed higher ORs for abnormal SUA levels, although some were not statistically significant.

**Table 2:** Results of factors associated between smoking behavior and serum uric acid.

Variables		Male				Female			
		Serum Uric Acid ≥7				Serum Uric Acid ≥6			
		OR	95% CI			OR	95% CI		
**Smoking Behavior**									
	Non-smoker	1.00				1.00			
	Ex-smoker	1.12	(0.97	-	1.30)	1.25	(0.91	-	1.71)
	Single smoker	1.03	(0.88	-	1.21)	1.68	(1.25	-	2.25)
	Dual smoker	1.43	(1.08	-	1.88)	1.88	(0.76	-	4.65)
**Age**									
	19–29	2.53	(1.89	-	3.40)	1.72	(1.15	-	2.57)
	30–39	2.32	(1.79	-	3.00)	1.50	(1.04	-	2.16)
	40–49	1.60	(1.26	-	2.04)	0.93	(0.65	-	1.31)
	50–59	0.97	(0.77	-	1.22)	1.13	(0.87	-	1.47)
	60–69	0.88	(0.70	-	1.10)	1.67	(1.31	-	2.14)
	≥70	1.00				1.00			
**Marital status**									
	Married	1.00				1.00			
	Single, widow	1.17	(1.00	-	1.37)	1.28	(1.04	-	1.57)
	Divorced, Separated	1.28	(0.94	-	1.75)	1.05	(0.77	-	1.43)
**Educational level**									
	Middle school or below	1.15	(0.95	-	1.39)	1.08	(0.80	-	1.46)
	High school	1.03	(0.90	-	1.18)	1.08	(0.87	-	1.33)
	College or over	1.00				1.00			
**Household income**									
	Low	1.00				1.00			
	Mid-low	1.10	(0.89	-	1.36)	1.01	(0.81	-	1.26)
	Mid-high	0.95	(0.77	-	1.17)	0.87	(0.68	-	1.10)
	High	1.03	(0.83	-	1.26)	1.10	(0.84	-	1.43)
**Region**									
	Metropolitan	1.00				1.00			
	Urban	1.01	(0.89	-	1.14)	1.02	(0.86	-	1.20)
	Rural	1.06	(0.89	-	1.26)	1.07	(0.87	-	1.33)
**Occupational categories**									
	White	1.13	(0.94	-	1.35)	0.85	(0.67	-	1.07)
	Pink	1.06	(0.86	-	1.31)	0.70	(0.55	-	0.88)
	Blue	0.90	(0.75	-	1.08)	0.73	(0.59	-	0.90)
	Inoccupation	1.00				1.00			
**BMI**									
	Underweight	1.00				1.00			
	Normal	2.02	(1.24	-	3.30)	3.77	(1.80	-	7.89)
	Overweight	3.95	(2.43	-	6.43)	10.30	(4.85	-	21.87)
**Alcohol consumption**									
	Yes	1.13	(0.96	-	1.33)	1.12	(0.96	-	1.32)
	No	1.00				1.00			
**Status of Hypertension**									
	Normal	1.00				1.00			
	Pre-Hypertension	1.43	(1.23	-	1.65)	1.52	(1.21	-	1.91)
	Hypertension	1.85	(1.59	-	2.15)	2.10	(1.67	-	2.64)
**Status of Diabetes**									
	Yes	0.63	(0.52	-	0.78)	1.40	(1.11	-	1.76)
	No	1.00				1.00			
**Status of Dyslipidemia**									
	Yes	1.14	(0.99	-	1.31)	1.16	(0.97	-	1.39)
	No	1.00				1.00			
**Year**									
	2016	1.00				1.00			
	2017	1.11	(0.93	-	1.33)	0.92	(0.70	-	1.20)
	2018	1.21	(1.00	-	1.46)	1.18	(0.92	-	1.51)
	2019	1.35	(1.12	-	1.62)	1.43	(1.12	-	1.83)
	2020	1.18	(0.99	-	1.41)	1.05	(0.81	-	1.36)

Table 3 demonstrates a subgroup analysis performed to evaluate the combined effect of smoking behavior, alcohol consumption, and hypertension on SUA levels. For men, alcohol consumption (OR, 1.43; 95% CI, 1.07–1.91) and hypertension (OR, 1.83; 95% CI, 1.01–3.32) among dual smokers had the strongest associations with SUA compared to those of non-smokers. For women, alcohol consumption (single smokers: OR, 1.68; 95% CI, 1.21–2.32), hypertension (dual smokers, OR: 16.99; 95% CI: 2.70–107.16), and dyslipidemia status (single smokers: OR, 1.83; 95% CI, 1.31–2.58) showed the strongest association with serum uric acid compared to those of non-smokers.

**Table 3:** Results of subgroup analysis stratified by independent variables.

Variables†			Serum Uric Acid Level												
			Smoking Behavior												
			Non-smoker	Ex-smoker				Single smoker				Dual smoker			
			OR	OR	95% CI			OR	95% CI			OR	95% CI		
**Male**	**BMI**														
		Underweight	1.00	2.61	(0.53	-	12.87)	0.50	(0.12	-	1.97)	*	*	*	*
		Normal	1.00	1.21	(0.98	-	1.50)	1.15	(0.91	-	1.46)	1.74	(1.11	-	2.71)
		Overweight	1.00	1.06	(0.87	-	1.28)	0.95	(0.77	-	1.17)	1.22	(0.85	-	1.75)
	**Alcohol consumption**														
		Yes	1.00	1.12	(0.96	-	1.31)	1.05	(0.88	-	1.24)	1.43	(1.07	-	1.91)
		No	1.00	1.10	(0.77	-	1.57)	0.89	(0.56	-	1.40)	1.65	(0.46	-	5.95)
	**Status of Hypertension**														
		Normal	1.00	1.05	(0.82	-	1.35)	1.11	(0.85	-	1.44)	1.38	(0.90	-	2.12)
		Pre-Hypertension	1.00	1.11	(0.87	-	1.40)	1.09	(0.85	-	1.39)	1.25	(0.80	-	1.96)
		Hypertension	1.00	1.22	(0.97	-	1.54)	0.94	(0.72	-	1.23)	1.83	(1.01	-	3.32)
	**Status of Diabetes**														
		Yes	1.00	1.01	(0.61	-	1.65)	0.55	(0.31	-	1.00)	1.64	(0.48	-	5.58)
		No	1.00	1.13	(0.97	-	1.31)	1.07	(0.91	-	1.26)	1.43	(1.07	-	1.90)
	**Status of Dyslipidemia**														
		Yes	1.00	1.29	(0.91	-	1.83)	1.21	(0.84	-	1.76)	1.01	(0.52	-	1.97)
		No	1.00	1.08	(0.92	-	1.27)	1.00	(0.83	-	1.19)	1.51	(1.11	-	2.06)
**Female**	**BMI**														
		Underweight	1.00	*	*	*	*	2.70	(0.19	-	38.94)	*	*	*	*
		Normal	1.00	1.68	(1.11	-	2.55)	2.14	(1.41	-	3.25)	2.37	(0.85	-	6.64)
		Overweight	1.00	0.88	(0.56	-	1.38)	1.20	(0.79	-	1.81)	1.52	(0.30	-	7.75)
	**Alcohol consumption**														
		Yes	1.00	1.21	(0.84	-	1.74)	1.68	(1.21	-	2.32)	2.05	(0.82	-	5.13)
		No	1.00	1.41	(0.79	-	2.52)	1.50	(0.72	-	3.09)	*	*	*	*
	**Status of Hypertension**														
		Normal	1.00	1.80	(1.17	-	2.76)	1.76	(1.15	-	2.68)	2.26	(0.73	-	7.03)
		Pre-Hypertension	1.00	0.66	(0.31	-	1.41)	1.53	(0.83	-	2.81)	0.32	(0.03	-	3.07)
		Hypertension	1.00	0.97	(0.59	-	1.62)	1.44	(0.87	-	2.40)	16.99	(2.70	-	107.16)
	**Status of Diabetes**														
		Yes	1.00	0.53	(0.17	-	1.60)	0.46	(0.15	-	1.47)	*	*	*	*
		No	1.00	1.33	(0.96	-	1.85)	1.90	(1.41	-	2.55)	2.00	(0.81	-	4.92)
	**Status of Dyslipidemia**														
		Yes	1.00	1.36	(0.94	-	1.95)	1.83	(1.31	-	2.58)	1.84	(0.68	-	4.95)
		No	1.00	0.95	(0.53	-	1.71)	1.37	(0.81	-	2.31)	2.16	(0.24	-	19.41)

† adjusted for all covariates

* Due to sparsity of the data, OR could not be calculated in the model

**Fig 1:**
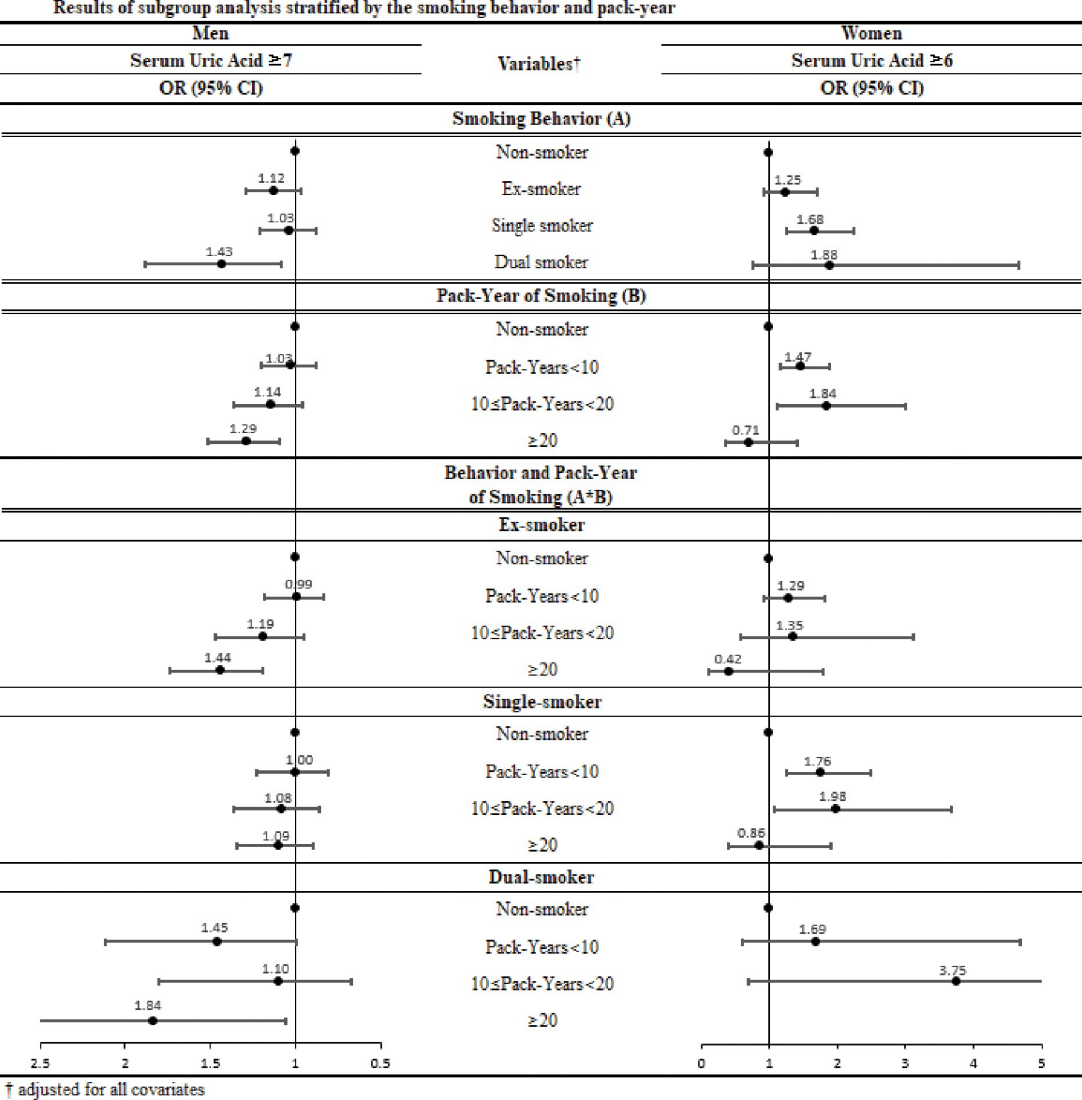
Results of subgroup analysis stratified by the smoking behavior and pack-year.

Fig 1 reveals the results of the subgroup analysis depicting changes in ORs according to the pack-years (number of cigarettes smoked and smoking period) smoked. The ORs tended to increase linearly as the pack-years increased. Specifically, male ex-smokers (OR, 1.44; 95% CI, 1.19–1.74) and dual smokers (OR, 1.84; 95% CI, 1.06–3.18) who had > 20 pack-years were more likely to have SUA levels ≥ 7 mg/dL than non-smokers. Female single smokers who had less than 10 pack-years (OR, 1.76; 95% CI, 1.25–2.48) and 10 to 20 pack-years (OR, 1.98; 95% CI, 1.07–3.66) were more likely to have SUA levels ≥ 6 mg/dL than non-smokers.

## Discussion

The World Health Organization has consistently emphasized the importance of quitting smoking and the dangers of smoking, which kills approximately eight million people every year [12]. However, the mechanism explaining how smoking increases SUA levels remains unclear. A study has revealed that current smokers with a BMI > 24.9 have an increased risk of gout over time [27]. This finding can be applied to single or dual smokers, as indirectly implied by the current results. Hyperuricemia is a major risk factor for metabolic syndrome that leads to the development of cardiovascular and cerebrovascular diseases [9]. Most patients with gout have obesity, hypertension, and hyperlipidemia [9, 25, 27]. Therefore, patients with gout may require proper management through smoking cessation to reduce this risk.

Based on this, the present study aimed to validate the association between SUA and various smoking behaviors, including dual smoking, single smoking, and ex-smoking, in a representative Korean adult population. We also conducted a subgroup analysis according to factors related to smoking and SUA, including BMI, alcohol consumption, hypertension, diabetes, and dyslipidemia status. Furthermore, we stratified smoking behavior according to pack-years smoked.

In this study, elevated SUA levels were observed in dual smokers compared to non-smokers. This relationship was especially strong among men who were dual smokers. A strong connection between elevated SUA levels and women who were single smokers was observed. Among men who had more than 20 pack-years, dual smokers and ex-smokers were more strongly associated with SUA. Among women who had less than 10 pack-years and 10 to 20 pack-years, single smoking was significantly associated with SUA. In general, the SUA level linearly increased with the pack-years. Overall, this study found a significant association among men but not among women, which could be considered a result of a recall bias in self-reported data owing to poor perception of women smoking in Korea [28]. The underreporting of women’s smoking is connected to social stigma, which conceals and masks smoking among women more so than men.

A previous study suggested that SUA was only associated with women, not men [24]. Furthermore, based on a previous study, our study considered dual smokers and found that SUA was related to both women and men. With the increase in the use of e-cigarettes, the risk perception of dual smokers has become important [1, 2]. Additionally, as the prevalence and incidence of gout increase [3, 4], research on the association between smoking and SUA is being actively conducted. The increasing effect of smoking on SUA has been observed globally [24, 29–31]. Moreover, one study found that male e-cigarette users have higher levels of SUA than non-smokers and conventional cigarette users [30]. In contrast, some studies suggested that smoking may lower SUA levels [32–34], which was explained by the antioxidant effect on ROS and free radicals produced by cigarettes [32]. No effect of reduction was reported to be found in a large study population considering the amount and duration of smoking [35, 36]. Although the association between smoking and SUA is controversial, generally, low SUA levels in smokers are associated with the depletion of antioxidants [24]. Therefore, these results are consistent with our findings on the adverse effects of e-cigarettes and dual smoking in our study population.

This study has certain limitations. First, it was a cross-sectional study. We found an association between smoking behavior and SUA; however, the causal relationship requires careful interpretation. Therefore, further research is needed to clarify the relationship of smoking behavior and SUA levels. Second, KNHANES data were collected as a self-report survey. Data on smoking behavior and health-related and socioeconomic variables might not have been accurately measured and might not be completely reliable. In particular, it may have resulted in recall bias with underestimated smoking behavior. Therefore, smoking behavior was evaluated on its own, and the exact smoking cessation status of ex-smokers was unclear. Future studies need to clear the smoking cessation period through measurement data to compensate for these limitations. Third, we aimed to consider the pack-years of all participants; however, owing to data limitations, we could not sufficiently reflect information on the pack-years of e-cigarettes. This may lead to uncertainty regarding the relationship between dual smokers and SUA. Therefore, further studies reflecting these data are needed. Fourth, e-cigarettes are more recent than conventional cigarettes, and a limited number of respondents smoked only e-cigarettes. Future research should consider each smoking behavior separately because single smokers who smoke only e-cigarettes were not considered. Finally, although we adjusted for many covariates that might have affected the results, residual confounding factors might not have been measured or considered in our analysis.

In contrast with the limitations, our study had several strengths. First, KNHANES conducted by the KDCA is nationally representative survey based on random cluster sampling, which is reliable and representative. Therefore, our results can be generalized to ordinary Korean adults. Second, blood samples were collected using standardized laboratory procedures, and SUA levels were measured to produce reliable and clear data. Third, few studies have evaluated the association between smoking behaviors, including e-cigarette use, dual smoking, and SUA. Therefore, this study is noteworthy in subgroup analysis by calculating pack-years and smoking behavior, such as dual smoking. In addition, the pack-years of ex-smokers and single and dual smokers were calculated and analyzed.

## Conclusion

Smoking behavior, particularly dual smoking, in the male population was associated with SUA. In addition, the higher the pack-years, the greater was the risk of high SUA levels. In particular, the risk of increased SUA levels, particularly in those who are dual smokers (> 20 pack-years), has been reported. Given these results, smoking is related to SUA, and dual and single smoking is harmful to health. These findings should provide the direction of research on the adverse effects of e-cigarettes and dual smoking in future studies and educate people regarding the risk. The current study findings may be significant given that many people believe using e-cigarettes to be safe smoking behaviors, and this could lead to dual smoking.

## References

[1] Engel B, Just J, Bleckwenn M, Weckbecker K. Treatment Options for Gout. Dtsch Arztebl Int. 2017;114: 215–222. doi: 10.3238/arztebl.2017.0215 28434436 PMC5624445

[2] Rock KL, Kataoka H, Lai JJ. Uric acid as a danger signal in gout and its comorbidities. Nat Rev Rheumatol. 2013;9: 13–23. doi: 10.1038/nrrheum.2012.143 22945591 PMC3648987

[3] Kuo CF, Grainge MJ, Zhang W, Doherty M. Global epidemiology of gout: prevalence, incidence and risk factors. Nat Rev Rheumatol. 2015;11: 649–662. doi: 10.1038/nrrheum.2015.91 26150127

[4] Singh JA, Edwards NL. Gout management and outcomes during the COVID-19 pandemic: a cross-sectional internet survey. Ther Adv Musculoskelet Dis. 2020;12: 1759720x20966124. doi: 10.1177/1759720X20966124 33133248 PMC7576903

[5] Simeunovic Ostojic M, Maas J. Anorexia nervosa and uric acid beyond gout: An idea worth researching. Int J Eat Disord. 2018;51: 97–101. doi: 10.1002/eat.22817 29314231

[6] Giordano C, Karasik O, King-Morris K, Asmar A. Uric Acid as a Marker of Kidney Disease: Review of the Current Literature. Dis Markers. 2015;2015: 382918. doi: 10.1155/2015/382918 26106252 PMC4461768

[7] Joo HJ, Kim GR, Choi DW, Joo JH, Park EC. Uric acid level and kidney function: a cross-sectional study of the Korean national health and nutrition examination survey (2016–2017). Sci Rep. 2020;10: 21672. doi: 10.1038/s41598-020-77702-x 33303792 PMC7730446

[8] Towiwat P, Li ZG. The association of vitamin C, alcohol, coffee, tea, milk and yogurt with uric acid and gout. Int J Rheum Dis. 2015;18: 495–501. doi: 10.1111/1756-185X.12622 26082349

[9] Borghi C, Agabiti-Rosei E, Johnson RJ, Kielstein JT, Lurbe E, Mancia G, et al. Hyperuricaemia and gout in cardiovascular, metabolic and kidney disease. Eur J Intern Med. 2020;80: 1–11. doi: 10.1016/j.ejim.2020.07.006 32739239

[10] Khanna D, Fitzgerald JD, Khanna PP, Bae S, Singh MK, Neogi T, et al. 2012 American College of Rheumatology guidelines for management of gout. Part 1: systematic nonpharmacologic and pharmacologic therapeutic approaches to hyperuricemia. Arthritis Care Res (Hoboken). 2012;64: 1431–1446. doi: 10.1002/acr.21772 23024028 PMC3683400

[11] Crawley WT, Jungels CG, Stenmark KR, Fini MA. U-shaped association of uric acid to overall-cause mortality and its impact on clinical management of hyperuricemia. Redox Biol. 2022;51: 102271. doi: 10.1016/j.redox.2022.102271 35228125 PMC8889273

[12] Organization WH. WHO Report on the Global Tobacco Epidemic, 2021: Addressing new and emerging products: World Health Organization; 2021.

[13] Callahan-Lyon P. Electronic cigarettes: human health effects. Tob Control. 2014;23 Suppl 2: ii36–40. doi: 10.1136/tobaccocontrol-2013-051470 24732161 PMC3995250

[14] Boyle RG, Richter S, St Claire AW. Defining adult e-cigarette prevalence: comparing a categorical definition with days of use. Tob Control. 2021;30: 530–533. doi: 10.1136/tobaccocontrol-2020-055641 32675251

[15] Layden JE, Ghinai I, Pray I, Kimball A, Layer M, Tenforde MW, et al. Pulmonary Illness Related to E-Cigarette Use in Illinois and Wisconsin—Final Report. N Engl J Med. 2020;382: 903–916. doi: 10.1056/NEJMoa1911614 31491072

[16] Choi DW, Jeon J, Lee SA, Han KT, Park EC, Jang SI. Association between Smoking Behavior Patterns and Glycated Hemoglobin Levels in a General Population. Int J Environ Res Public Health. 2018;15. doi: 10.3390/ijerph15102260 30332732 PMC6210515

[17] Jeong SH, Jang BN, Kim SH, Jang SI, Park EC. Investigation of the Association between Smoking Behavior and Metabolic Syndrome Using Lipid Accumulation Product Index among South Korean Adults. Int J Environ Res Public Health. 2021;18. doi: 10.3390/ijerph18084151 33919954 PMC8070901

[18] Cherian SV, Kumar A, Estrada YMRM. E-Cigarette or Vaping Product-Associated Lung Injury: A Review. Am J Med. 2020;133: 657–663. doi: 10.1016/j.amjmed.2020.02.004 32179055

[19] Glynos C, Bibli SI, Katsaounou P, Pavlidou A, Magkou C, Karavana V, et al. Comparison of the effects of e-cigarette vapor with cigarette smoke on lung function and inflammation in mice. Am J Physiol Lung Cell Mol Physiol. 2018;315: L662–l672. doi: 10.1152/ajplung.00389.2017 30091379

[20] Kim CY, Paek YJ, Seo HG, Cheong YS, Lee CM, Park SM, et al. Dual use of electronic and conventional cigarettes is associated with higher cardiovascular risk factors in Korean men. Sci Rep. 2020;10: 5612. doi: 10.1038/s41598-020-62545-3 32221375 PMC7101350

[21] Tomar SL, Alpert HR, Connolly GN. Patterns of dual use of cigarettes and smokeless tobacco among US males: findings from national surveys. Tob Control. 2010;19: 104–109. doi: 10.1136/tc.2009.031070 20008157 PMC2989167

[22] Piper ME, Baker TB, Benowitz NL, Kobinsky KH, Jorenby DE. Dual Users Compared to Smokers: Demographics, Dependence, and Biomarkers. Nicotine Tob Res. 2019;21: 1279–1284. doi: 10.1093/ntr/nty231 30365010 PMC7182769

[23] Choi SH, Ling J, Noonan D, Kim W. Smoking behavior and social contexts associated with smoking among dual-smoker couples. Public Health Nurs. 2020;37: 161–168. doi: 10.1111/phn.12686 31724240

[24] Kim SK, Choe JY. Association between smoking and serum uric acid in Korean population: Data from the seventh Korea national health and nutrition examination survey 2016. Medicine (Baltimore). 2019;98: e14507. doi: 10.1097/MD.0000000000014507 30762781 PMC6407981

[25] Xu N, Han X, Zhang Y, Huang X, Zhu W, Shen M, et al. Clinical features of gout in adult patients with type Ia glycogen storage disease: a single-centre retrospective study and a review of literature. Arthritis Res Ther. 2022;24: 58. doi: 10.1186/s13075-021-02706-5 35219330 PMC8881853

[26] Oh SS, Jang JE, Lee DW, Park EC, Jang SI. Cigarette type or smoking history: Which has a greater impact on the metabolic syndrome and its components? Sci Rep. 2020;10: 10467. doi: 10.1038/s41598-020-67524-2 32591636 PMC7319978

[27] Kurniasari MD, Karwur FF, Rayanti RE, Dharmana E, Rias YA, Chou KR, et al. Second-Hand Smoke and Its Synergistic Effect with a Body-Mass Index of >24.9 kg/m(2) Increase the Risk of Gout Arthritis in Indonesia. Int J Environ Res Public Health. 2021;18. doi: 10.3390/ijerph18084324 33921811 PMC8073587

[28] Kang HG, Kwon KH, Lee IW, Jung B, Park EC, Jang SI. Biochemically-verified smoking rate trends and factors associated with inaccurate self-reporting of smoking habits in Korean women. Asian Pac J Cancer Prev. 2013;14: 6807–6812. doi: 10.7314/apjcp.2013.14.11.6807 24377610

[29] Fukuhara A, Saito J, Sato S, Saito K, Fukuhara N, Tanino Y, et al. The association between risk of airflow limitation and serum uric acid measured at medical health check-ups. Int J Chron Obstruct Pulmon Dis. 2017;12: 1213–1219. doi: 10.2147/COPD.S126249 28458533 PMC5402911

[30] Moon J, Lee H, Kong M, Kim H, Oh Y. Association Between Electronic Cigarette Use and Levels of High-Sensitivity C-Reactive Protein and Uric Acid. Asia Pac J Public Health. 2020;32: 35–41. doi: 10.1177/1010539519899777 31955613

[31] Golli NE, Jrad-Lamine A, Neffati H, Dkhili H, Rahali D, Dallagi Y, et al. Impact of e-cigarette refill liquid exposure on rat kidney. Regul Toxicol Pharmacol. 2016;77: 109–116. doi: 10.1016/j.yrtph.2016.02.012 26925498

[32] Tsuchiya M, Asada A, Kasahara E, Sato EF, Shindo M, Inoue M. Smoking a single cigarette rapidly reduces combined concentrations of nitrate and nitrite and concentrations of antioxidants in plasma. Circulation. 2002;105: 1155–1157. doi: 10.1161/hc1002.105935 11889006

[33] Haj Mouhamed D, Ezzaher A, Neffati F, Douki W, Gaha L, Najjar MF. Effect of cigarette smoking on plasma uric acid concentrations. Environ Health Prev Med. 2011;16: 307–312. doi: 10.1007/s12199-010-0198-2 21431788 PMC3156839

[34] Hanna BE, Hamed JM, Touhala LM. Serum uric Acid in smokers. Oman Med J. 2008;23: 269–274 doi: 10.1161/hc1002.105935 22334840 PMC3273920

[35] Okubo Y, Miyamoto T, Suwazono Y, Kobayashi E, Nogawa K. An association between smoking habits and blood pressure in normotensive Japanese men. J Hum Hypertens. 2002;16: 91–96. doi: 10.1038/sj.jhh.1001303 11850765

[36] Gordon T, Kannel WB, Dawber TR, McGee D. Changes associated with quitting cigarette smoking: the Framingham Study. Am Heart J. 1975;90: 322–328. doi: 10.1016/0002-8703(75)90320-8 1163424

